# The Efficacy and Safety of a Single Treatment of High‐Intensity, High‐Frequency, Non‐Focused Ultrasound Parallel Beams for Facial Acne Scars in Asian Patients: A Preliminary Study

**DOI:** 10.1111/jocd.70134

**Published:** 2025-03-25

**Authors:** Kanjana Boonchoo, Wichai Hongcharu

**Affiliations:** ^1^ Dr. Wichai Clinic Bangkok Thailand

**Keywords:** acne scar, high‐intensity fractional ultrasound, high‐intensity parallel beam ultrasound, non‐focused ultrasound, noninvasive skin tightening, skin tightening, sofwave, synchronous ultrasound parallel beam technology

## Abstract

**Background:**

Acne vulgaris is a common inflammatory disease that often leads to changes in skin texture. While cosmetic improvement of acne scars is achievable, complete restoration remains challenging. One promising treatment is synchronous ultrasound (US) parallel beam technology, a noninvasive device that targets the mid‐dermis while preserving the epidermis. This technology induces controlled thermal injury, stimulating neo‐collagenesis and neo‐elastinogenesis, which contribute to the improvement of atrophic acne scars.

**Objective:**

To assess the efficacy and safety of high‐intensity, high‐frequency, non‐focused US parallel beams for the treatment of facial acne scars in Asian patients.

**Materials and Methods:**

Fourteen subjects, aged 24–55 years, underwent a single US treatment and were evaluated for improvement in facial acne scars in a retrospective study. The follow‐up period extended up to 8 months after treatment. Pre‐ and post‐treatment photographs were assessed by a blinded dermatologist using the physician global aesthetic improvement scale (PGAIS) and the acne scar severity (ASS) scale. Scar volume was measured using the Antera 3D software.

Pain perception was assessed immediately after treatment using an 11‐point scale. Any safety concerns were recorded and examined.

**Results:**

Fourteen Thai subjects, with a mean age of 43 years, showed significant improvement in depression volumes (small, medium, and large) at follow‐up visits compared to baseline. From PGAIS results, all treated areas showed improvement. The mean pain score was 3.94. No adverse events were reported.

**Conclusion:**

Synchronous US parallel beam technology appears to be a promising and safe option for treating acne scars in Asian patients.

## 1 Introduction

Acne vulgaris is a common inflammatory disease of the pilosebaceous unit, often resulting in skin texture changes and hyperpigmentation [1]. Excessive sebum production is a major contributor to acne development [1]. The accumulation of sebum, along with the keratinous material shed from the skin, clogs the hair follicle, leading to the formation of a comedo. When exposed to an infectious environment, particularly with *P. acnes* bacteria, the inflammatory process is exacerbated [1]. Acne scars have significant implications for the quality of life of affected individuals, often leading to mental health issues such as depression, embarrassment, and other social‐related impairments [2]. Early intervention is essential to prevent further disease progression and severe implications. Traditional remedies and conventional treatments for acne include plant‐based extracts, topical retinoids, topical and systemic antimicrobials, and other systemic medications [1, 3, 4].

**FIGURE 1:**
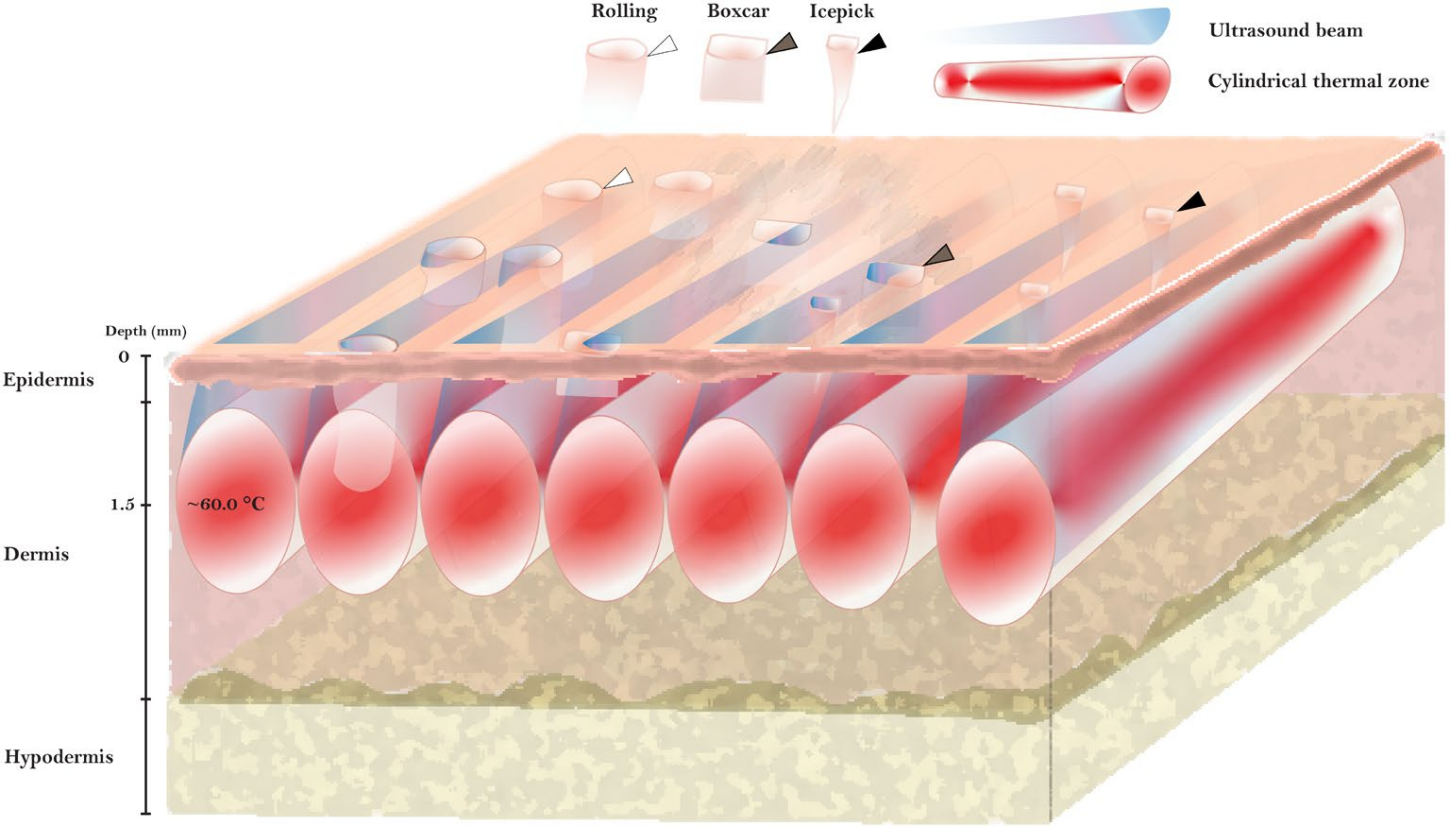
Ultrasound parallel beam thermal effect. Schematic representation of the synchronous ultrasound parallel beam (SUPERB) biophysical effect during its application to human skin. Atrophic acne scar types [5] are indicated in the figure. The high‐intensity, high‐frequency ultrasound parallel beams generated by multiple synchronized transducers penetrate to the mid‐dermis at depths of 0.5–2 mm and consequently create multiple unique cylindrical volumetric thermal zones. The temperature within the thermal zone varies from 60°C to 70°C leading to partial denaturation of collagen and elastin. (Illustration was created by SofWave Medical Ltd. using Adobe Illustrator software V28.6).

Atrophic acne scars are significantly more common than keloid and hypertrophic scars [5]. These scars develop as a result of inflammatory acne lesions that cause collagen loss and inadequate extracellular matrix (ECM) remodeling, leading to depressed areas in the skin. Atrophic acne scars are subclassified into ice pick, boxcar, and rolling scars [5] (Figure 1). Various treatment modalities have been employed to restore lost collagen and improve the appearance of scars. These treatments are categorized into topical therapies, procedural and energy‐based device (EBD) interventions, and surgical techniques [5, 6, 7, 8]. Each treatment option carries its own risks and side effects, which can vary depending on the treatment type, skin type, and patient lifestyle. Surgical and minimally invasive techniques, such as subcision, punch excision, injectable fillers, and fat transfer, have proven effective for treating acne scars. However, they are associated with potential side effects like bruising, swelling, pain, and an increased risk of infection [5, 9].

Ablative procedures, including dermabrasion and ablative laser treatments, carry a higher risk of complications, such as bacterial and herpes infections, persistent erythema, hyperpigmentation, and scarring. Specifically, scarring following CO_2_ laser therapy may occur due to overtreatment [5, 6, 7].

Non‐ablative lasers, such as the 1064 nm Nd: YAG and 1540 nm erbium glass lasers, do not ablate tissue but instead deliver a controlled thermal injury to the dermis, stimulating collagen production, which leads to a reduction in scar volume [5, 6, 7].

Although non‐ablative lasers are considered a safer alternative and are increasingly preferred for treating facial wrinkles and acne scars, owing to their minimal downtime and lower risk of complications [5, 6, 7], they still carry a risk of post‐inflammatory hyperpigmentation (PIH), especially in patients with darker skin types. A correlation between prolonged post‐treatment erythema and higher energy settings, as well as between PIH and higher density settings, has been reported in Asian patients when using a non‐ablative 1550 nm fractional laser system [9].

Unlike lasers and light‐based energy treatments, radiofrequency (RF) and focused ultrasound (US) offer safer options for all skin types with minimal downtime. Both technologies deliver energy into the dermis and aim to stimulate collagen production and ECM remodeling to improve skin texture. Microneedle RF combines microneedles with RF energy, creating micro‐injuries in the skin and delivering heat into its layers at adjustable depths, depending on the needle length, energy settings, and acne scar type. Non‐insulated microneedle RF offers an advantage over insulated needles, as it emits energy from both the tip and shaft, covering a broader treatment area [10, 11, 12].

Focused US, including micro‐ and macro‐focused US, utilizes US waves to heat deep layers of the skin without compromising the epidermal layer, stimulating collagen remodeling and promoting skin tightening [11, 12]. The transducers in US devices generate waves that target varying skin depths. For instance, at a depth of 4.5 mm, the US waves reach the superficial musculoaponeurotic system (SMAS), resulting in collagen denaturation and a tissue tightening effect [12]. At 1.5 mm, the waves target the mid‐dermis, which is rich in collagen and elastin fibers [13], stimulating neo‐collagenesis and improving skin laxity and texture [11].

Despite their effectiveness, earlier focused US devices were associated with complications such as PIH, pain, nerve injury, and skin burns [12, 14]. These issues were often attributed to insufficient energy discharge. To address these limitations, a new high‐intensity US device was developed by SofWave Medical Ltd. The device has been FDA‐cleared for wrinkle reduction and lifting treatments of the eyebrows, neck, and submental areas [15]. This device utilizes Synchronous Ultrasound Parallel Beam (SUPERB) Technology, delivering energy at a depth of 1.5 mm with an intensity of up to 5 J. Its active integrated cooling system, along with a real‐time skin temperature monitor, effectively prevents thermal damage to the epidermis (Figure 1). With these technological advancements, we propose a retrospective preliminary study to assess the efficacy and safety of a single treatment with high‐intensity, high‐frequency, non‐focused US parallel beams for facial acne scars in Asian patients.

## 2 Materials and Methods

### 2.1 Overview

**FIGURE 2:**
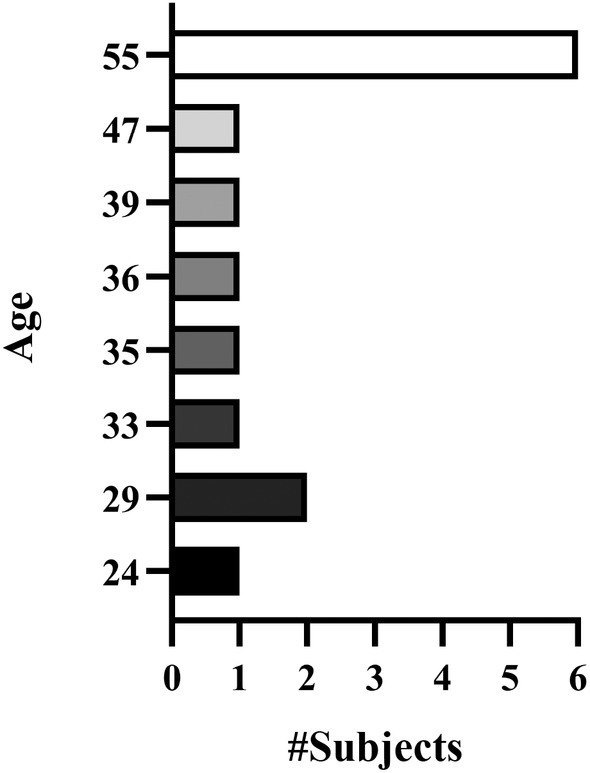
Subjects age range. Fourteen subjects were included in the study, five of whom were males. Among the male subjects, four were 55 years old and one was 29. The remaining nine subjects were females, with the youngest being a 24‐year‐old female and the oldest also 55 years old.

To assess the safety and efficacy of the high‐intensity, high‐frequency, non‐focused parallel ultrasonic device (SofWave Medical Ltd., Yokneam Ilit, Israel) for improving facial acne scars, data were retrospectively collected from subjects with mild to moderate acne scars located on the cheeks and/or temples. The study was conducted in accordance with the Declaration of Helsinki. Fourteen [14] Thai subjects, (5 males and 9 females) aged 24 to 55 years (Figure 2), with Fitzpatrick skin types II to IV, were enrolled in this preliminary observational retrospective study at Dr. Wichai's clinic in Bangkok, Thailand, between November 2021 and January 2024. Signed informed consent and photo consent were obtained from all subjects.

### 2.2 Inclusion and Exclusion Criteria

Inclusion criteria were males and females aged 22–80 years old who had received a single SofWave treatment for skin tightening and had complete medical records, including at least one follow‐up visit with digital imaging and 3D imaging data.

Subjects were excluded from the study if they had a history of any cosmetic treatments in the intended treatment area prior to the follow‐up session. Additional exclusion criteria included patients who were lost to follow‐up for more than 12 months; had any conditions that might affect the outcome; severe solar elastosis; a history of chronic drug or alcohol abuse; usage of anticoagulant or antiplatelet drugs; were pregnant or planning to become pregnant; were breastfeeding or had given birth during the follow‐up period; or had any other condition that might make participation unsafe as per the principal investigator's decision.

### 2.3 Treatment

Subjects underwent a single SofWave treatment on the entire face and attended follow‐up visits up to 8 months after the treatment. Before treatment, subjects were instructed to cleanse and dry their face. Ultrasonic gel was applied to the treatment area to serve as a coupling medium, and air cooling was used for pain relief during the procedure. No topical anesthesia was applied.

The high‐intensity, high‐frequency parallel beam transducers operated at 10–12 MHz with a focal depth of 1.5 mm and were applied to the entire face, including the cheeks and/or temples.

Treatment parameters included a pulse duration of 5 s; post cooling time of 1–3 s, and an energy range of 3.4 to 4.2 J (Table 1) depending on the characteristics of the treated area and the subject's pain tolerance. The procedure lasted from 45 to 60 min.

**TABLE 1:** Treatment program.

Area	Energy (J)/pulse	Total pulses/side	Total passes
Forehead	3.4	40–50	1
Periorbital	3.4–4.2	10	2
Cheek	3.8–4.2	100–120	2–3

After treatment, the ultrasonic gel was removed, and the treated area was cleaned. Subjects were informed that erythema and edema might develop in the treated area but were expected to resolve spontaneously. All participants were instructed to notify the clinic if any side effects occurred after the procedure for further dermatological examination.

Standardized digital photographs and 3D images were taken by the same independent operator using a Sony A9 camera and the Antera 3D camera (Antera Pro v2.8.5; Miravex Limited), respectively, at baseline (pretreatment) and during follow‐up visits (up to 8‐months post‐treatment). These images were used for retrospective analysis of 16 facial areas using the Antera 3D software.

The Antera 3D images were analyzed at different lateral sizes, measuring the volume of depressions at three levels: small (0.1–1 mm), medium (0.1–2 mm), and large (0.1–3 mm).

The baseline value was considered 100%, and the post‐treatment values were compared to this baseline. Any post‐treatment value lower than 100% was considered an improvement, and any higher value indicated worsening.

To minimize operator dependency and lighting variations, the same photographer was used throughout the study, with all images captured under consistent conditions. The baseline image of each patient served as a reference to ensure consistent positioning and angle. The photographer in this study was not involved in the treatments or post‐treatment assessments.

Immediately after treatment, subjects were asked to rank their pain level during the treatment using an 11‐point visual analog scale (0 = no pain while 10 = worst pain imaginable).

Post‐treatment responses and any adverse effects (AE) were assessed and recorded by the investigators throughout the study.

ASS (0 = absent; 1 = mild; 2 = moderate; 3 = severe) and PGAIS (−1 = worse; 0 = no change; 1 = improved; 2 = marked improvement; 3 = very much improved) were used to assess improvement based on the 3D and/or digital images and were evaluated by a blinded dermatologist.

Subjects resumed normal life activities with no restrictions.

### 2.4 Statistical Analysis

Statistical analysis and graphical visualizations were performed using IBM SPSS, GraphPad Prism 10, and Adobe Illustrator 2024 software. The Wilcoxon paired test and Kruskal–Wallis test were used, with results interpreted at a 5% significance level (Key: not significant (ns) > 0.05, * ≤ 0.05, ** ≤ 0.01). Continuous variables were reported as mean ± standard deviation (SD).

### 2.5 Device Patents

The novel ultrasound technology is comprehensively covered by several patents, with the most relevant being those for the ultrasound transducer and system (WO2017212489), fat tissue treatment (WO2020026253), and skin treatment system (WO2021111450). Detailed documentation can be found on the World Intellectual Property Organization (WIPO) official website (wipo.int).

## 3 Results

### 3.1 Subject Demographics

Fourteen [14] Thai subjects (9 females, 5 males) with a mean age of 43 years (R: 24–55 years) (Figure 2) underwent a single treatment on the entire face. Acne scars were analyzed on the cheek [12] and temple [4] areas. One subject (7.1%) was classified as Fitzpatrick skin type (ST) II, 9 subjects (64.3%) ST III, and 4 subjects (28.6%) were classified as ST IV. Regarding acne scars severity at baseline, as assessed using the ASS scale, 2 subjects (14.3%) had grade 1 scars, 6 subjects (42.9%) had grade 2 scars, and the remaining 6 subjects (42.9%) had grade 3 scars (Figure 5C).

### 3.2 Treatment Parameters

The treatment area included the entire face. Depression volumes were analyzed at baseline and follow‐up visits across 16 facial areas, including the cheeks and temples. The amount of energy applied to each area ranged between 3.4 and 4.2 J, with 40–120 pulses per side in 1–3 passes (Table 1).

### 3.3 Clinical Results and Ratings

**FIGURE 3:**
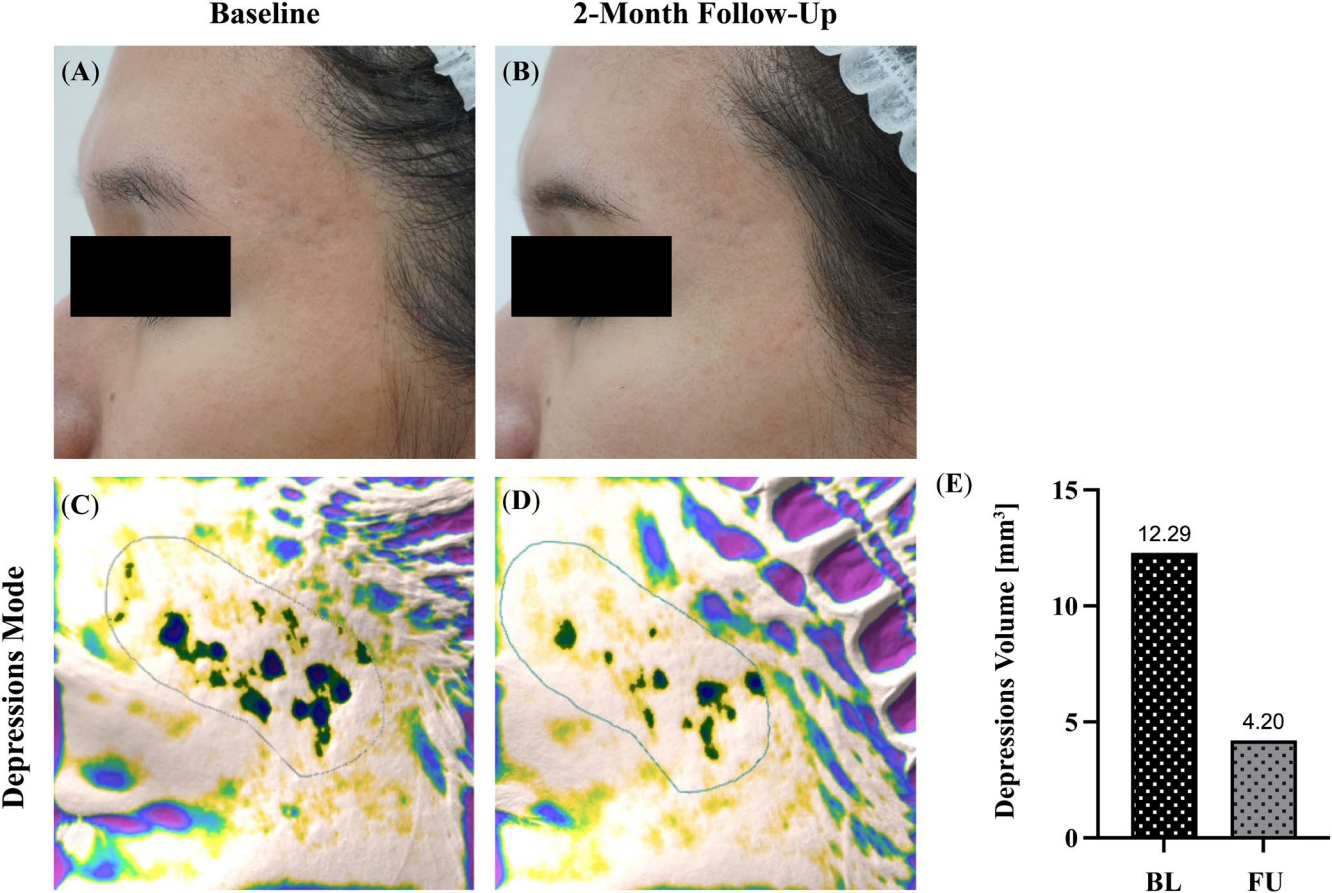
Antera 3D large depressions volume example. Subject 01, female, 33 years old. (A, B) Photograph of subject's left temple area at baseline (A) and 2‐month follow‐up (B) visits. (C, D) Antera 3D scan of subject's left temple area at baseline (C) and 2‐month follow‐up (D) visits. (A–D) Images of the same area and orientation. Acne scar depressions are encircled (C, D). (E) Subject 01 depressions volume at baseline (BL) and follow‐up (FU) were 12.29 mm^3^ and 4.20 mm^3^, respectively, reflecting a visible 65.8% improvement.

**FIGURE 4:**
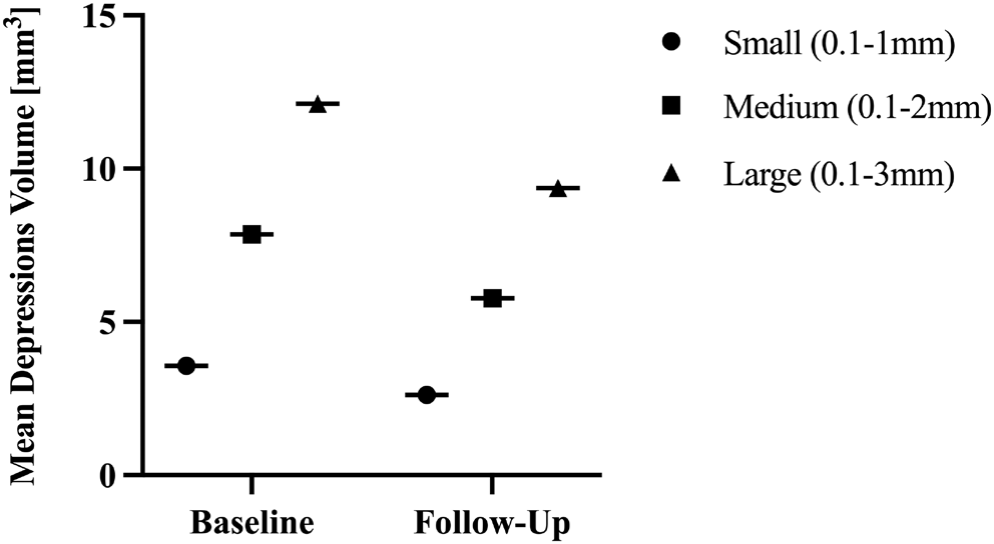
Mean depression volume change. Mean depression volume of small, medium, and large depressions at baseline and follow‐up visits. The mean change was significant for all depths (< 0.001). Mean values are described in Table 2.

Subjects had varying follow‐up times, with the longest follow‐up extending to 8 months and an average follow‐up time of 3.6 months post‐treatment. The Antera 3D analysis (see example in Figure 3) revealed a significant reduction in the mean depression volume in all three assessed depths (Figure 4), (Table 2).

**TABLE 2:** Mean Antera 3D depression measurements summary.

	Small depressions (0.1–1 mm)		Medium depressions (0.1–2 mm)		Large depressions (0.1–3 mm)	
	Baseline	Follow‐up	Baseline	Follow‐up	Baseline	Follow‐up
Mean	3.57	2.62	7.86	5.77	12.12	9.37
S.D.	2.47	1.87	4.79	4.03	7.45	7.11
Minimum	0.7	0.47	1.84	1.26	2.55	0.76
Maximum	9.06	6.4	17.34	14.15	27.01	23.49
Treated areas	16	16	15	15	14	14
*p* ^a^	0.0004***		0.0008***		0.001***	

^a^ Wilcoxon signed rank test for paired data. *** *p* ≤ 0.001.

Small and medium depressions were reduced by an average of 27% (*p*‐value: 0.0004 and 0.0008, respectively) when compared to baseline and follow‐up values. Large depressions showed a reduction of 23% on average (*p*‐value = 0.001).

No statistically significant differences were observed between the three depression depths.

**FIGURE 5:**
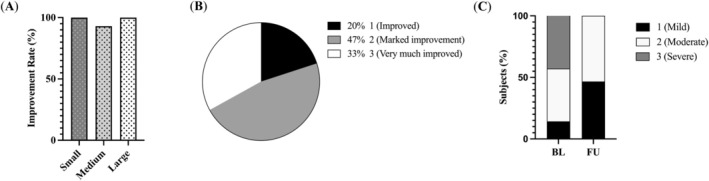
Acne scars improvement. (A) Improvement rate of small (100%), medium (93%), and large (100%) depressions. (B) Physician Global Aesthetic Improvement Scale results (% subjects). (C) Acne scars severity score results at baseline (BL) and follow‐up (FU) visits. Baseline portions were 14% (mild), 43% (moderate), and 43% (severe) whereas follow‐up values split to 47% (mild) and 53% (moderate).

The improvement rate (Figure 5A) was 100% for both small and large depressions. However, for medium depressions, the improvement rate was 93% due to one subject who was treated on the cheeks. This subject showed an improvement in small depressions (0.83mm^3^ and 0.68mm^3^ at baseline and follow‐up, respectively) but no improvement in medium depressions (7.58mm^3^ and 7.59mm^3^ at baseline and follow‐up, respectively). This subject was not assessed for large depressions using the Antera 3D software.

According to the PGAIS results (Figure 5B), all treated areas showed an improvement, 47% were graded as “marked improvement” and 33% as “very much improved.”

The ASS assessment showed that 64% of treated areas improved. Notably, all areas that were graded as “severe” at baseline showed improvement, with all being regraded as “mild” or “moderate” at the follow‐up visit (Figure 5C).

### 3.4 Safety and Pain

The mean pain ± standard deviation level during treatment was 3.94 ± 0.77 on the 11‐point scale. Almost half of the subjects (44%) reported a pain level of 4, while 31% and 25% of subjects reported pain levels of 3 and 5, respectively. No adverse events were observed during the study period.

## 4 Discussion

Acne vulgaris affects an estimated 9.4% of the global population, making it the eighth most prevalent disease worldwide [4, 16]. The symptoms of acne are often frustrating, and its long‐term effects, including scarring and the financial burden of treatment, can significantly impact patients' quality of life [4, 17]. Koo [2] described that acne not only causes skin irregularities but also impacts psychological and social well‐being, often leading to depression, low self‐confidence, anger, and social impairment. Due to the high incidence of acne scarring and its long‐term implications [2, 5], early treatment of active acne is crucial in preventing the development of scars. Chilicka et al. [18] outlined several cosmetic methods that can help reduce skin eruptions and acne scars. Among the available options are cosmetic acids including salicylic acid, an organic compound commonly used to treat noninflammatory lesions due to its milder effect; glycolic acid, which provides a moisturizing effect at lower concentrations and exfoliation at higher concentrations; azelaic acid, which inhibits the growth of *C. acnes*; and trichloroacetic acid (TCA), which can improve acne scars and skin discoloration [4, 6, 18]. Microdermabrasion, oxybrasion, and hydrogen purification are also effective approaches for mechanically exfoliating the epidermis to treat acne, and in some cases, can be combined with cosmetic acids [4, 18]. The latest acne treatment guidelines from the American Academy of Dermatology recommend a range of treatments, from topical therapies such as retinoids and antibiotics to systemic treatments like isotretinoin and oral antibiotics [4]. For patients who cannot tolerate the side effects of conventional therapies such as the teratogenicity associated with isotretinoin or the cutaneous irritation caused by azelaic acid, traditional remedies may offer an effective alternative with a lower risk of AE. Herbal and plant‐based extracts have been reported to improve acne with fewer side effects [3]. Sharif et al. [19] demonstrated the beneficial effects of the *M. hamburg* grape, which contains organic acids, mineral salts, enzymes, vitamins, carotenoids, and polyphenols known for their antioxidant properties. The application of a 2% *M. hamburg* grape seed extract on human cheek skin twice daily for eight weeks was shown to safely improve acne, hyperpigmentation, skin elasticity, and reduce skin sebum content [19].

Unlike treatments for active acne, there are no standardized guidelines for treating acne scars. However, multiple studies have demonstrated the effectiveness of EBDs, both invasive and noninvasive, in improving acne scars [7, 10, 11, 12, 20]. Ablative procedures, such as microneedling and ablative lasers, show promising results although they carry a higher risk of complications, including discomfort, scarring, infections, and hyperpigmentation [5, 6, 7, 8, 9, 10, 11, 12, 20]. In particular, aggressive CO_2_ laser treatments have been associated with scarring [6]. To mitigate these risks, non‐ablative lasers have been introduced for skin resurfacing, but similar side effects have still been reported [7, 12, 20]. Rho [9] observed a correlation between prolonged post‐treatment erythema and higher energy settings when using a non‐ablative 1550 nm fractional laser system in the Korean population. PIH was also more pronounced in patients treated with higher‐density settings, and acne flares were noted in relation to higher energy levels [9].

In contrast to laser treatments, RF and US technologies are not affected by chromophores, such as melanin, thus reducing the potential risks to the epidermal layer. In a study by Taub and Garretson [21], the use of sublative fractional bipolar RF and bipolar RF combined with a diode laser resulted in at least moderate improvement in acne scars in 75% of subjects, with a 69% satisfaction rate after five treatment sessions. Other studies [22, 23] have also reported improvement in acne scars following treatment with fractional RF devices, with high satisfaction rates. However, side effects such as prolonged erythema, hyperpigmentation, and crusting were still observed [22, 23]. Phothong et al. [23] found that higher energy and density settings with the bipolar fractional RF device led to higher rates of prolonged erythema and PIH, respectively.

Several US devices, known for their skin‐tightening effect, have also been used to treat atrophic acne scars due to their efficacy in collagen stimulation and minimal epidermal injury.

The accumulated ultrasonic energy in the dermis causes partial collagen denaturation, triggering a complex molecular process that stimulates the production of collagen and elastin, as well as the remodeling of the ECM [7, 11, 12].

Maas and Joseph [24] demonstrated the substantial effectiveness of micro‐focused US with visualization (MFU‐V) in treating acne scars, with all subjects showing improvement as determined by three blinded reviewers and high satisfaction rates. However, their study involved multiple treatment sessions at focal depths of 3.0 mm and 1.5 mm [24].

Similar to our study, Wang et al. [15] demonstrated the effectiveness and safety of high‐intensity, high‐frequency parallel US beams for wrinkle treatment. The device handpiece is equipped with seven parallel transducers that deliver energy to the mid‐dermis and create 3D cylindrical thermal zones (Figure 1) [15]. The coagulation zone is rich in collagen and elastin and ranges from 0.5 to 2 mm, with a central focus at 1.5 mm [13, 15]. The size of the thermal zone varies based on the energy level, and all seven transducers activate simultaneously with each pulse to ensure extensive tissue coverage. In vivo histologic analysis shows that performing two passes over the treatment area with a 50% overlap effectively covers approximately 28% of the mid‐dermal layer [15].

Our study expands on these findings by demonstrating that this novel technology is also effective for treating acne scars. Both quantitative (Antera 3D analysis) and qualitative (blinded dermatologist assessment) methods showed significant improvements in the appearance of acne scars from baseline to follow‐up, across various depression depths including small (0.1–1 mm), medium (0.1–2 mm), and large (0.1–3 mm) depressions.

Interestingly, no significant difference was found among the three depression depths, indicating a consistent treatment effect at all assessed depths.

The blinded dermatologist assessment corroborated the quantitative results, with the PGAIS showing 100% improvement in treated areas, 47% of which were graded as “marked improvement” and 33% as “very much improved.” According to the ASS scale, 64% of treated areas showed improvement, with all areas that were graded as “severe” at baseline improving to “mild” or “moderate” at follow‐up.

Notably, these impressive results were achieved after a single high‐intensity, high‐frequency US treatment, unlike other studies using MFU‐V and bipolar fractional RF, which frequently required multiple treatment sessions [21, 22, 23, 24]. This raises the question of whether multiple high‐intensity, high‐frequency US treatment sessions would lead to even greater improvements in acne scars.

Our study found no adverse effects (AE) during treatment, even with energy levels up to 4.2 J. In contrast, previous studies using US‐based devices with focal depths of 1.5 mm, 3 mm, and 4.5 mm transducers have reported AEs such as fat atrophy, skin blistering, focal bruising, PIH, and facial edema [14]. The procedure was well tolerated without the need for topical anesthesia, with a mean pain score of 3.94 ± 0.77 on an 11‐point scale.

No cases of facial fat loss or nerve damage were reported in our study, suggesting that the procedure is both time‐efficient and has a high safety profile.

Our findings suggest that high‐intensity, high‐frequency US is a safe and effective option for treating atrophic acne scars in darker skin types. It is particularly suitable for patients seeking significant improvement after a single session and those who prefer minimal downtime. This study is a retrospective analysis conducted on patients initially seeking facial skin tightening in 2021 before the device received FDA approval for acne scar treatment. As such, the whole face was treated and the improvement in acne scars appearance was an accidental finding, later evaluated retrospectively. Hence, future studies should apply the device to target acne scar areas to better assess its effectiveness for this specific purpose. This approach, combined with histological evaluation, will help clarify whether the observed improvement results from new collagen production beneath the scar rather than just adjacent skin tightening effects. Additionally, further studies are needed, as the longest follow‐up in our study was only eight months, with an average follow‐up time of 3.6 months.

We also observed impressive results on boxcar‐type scars in the temple area (Figure 3). Temple acne scars are notoriously difficult to treat due to the loss of soft tissue volume in this region [25].

This study has some limitations, and further research is recommended to compare the outcomes of different US devices at varying depths while addressing safety considerations. The small cohort of 14 subjects limits the study's statistical power, and a larger sample size in future research would improve generalizability. While the focus on Fitzpatrick skin types II–IV aligns with the target demographic, it restricts applicability to individuals with other skin types and ethnic groups. Additionally, the average follow‐up period of 3.6 months is relatively short for assessing long‐term scar improvement and potential delayed complications. Extending the follow‐up period to 12–18 months would enable a more comprehensive evaluation of treatment durability and late‐onset side effects.

A prospective, randomized controlled trial (RCT) would provide stronger evidence of the efficacy and safety profiles of this treatment. Furthermore, incorporating biopsy data to confirm neo‐collagenesis would enhance the study's biological validity.

In conclusion, acne scars remain challenging to treat, but several effective and safe options, including topical treatments, EBDs, and surgical techniques, can be tailored to individual patients. This study introduces a novel high‐intensity, high‐frequency, parallel beam US device as a promising and safe alternative for treating atrophic acne scars, particularly in Asian skin types.

## Ethics Statement

The study was conducted in accordance with the Declaration of Helsinki.

## Consent

Written and informed consent was obtained from all the participants of the study.

## Conflicts of Interest

The authors declare no conflicts of interest.

## Data Availability

The data that support the findings of this study are available on request from the corresponding author. The data are not publicly available due to privacy or ethical restrictions.
